# Safety injections of nuclear medicine radiotracers: towards a new modality for a real-time detection of extravasation events and ^18^F-FDG SUV data correction

**DOI:** 10.1186/s40658-023-00556-5

**Published:** 2023-05-23

**Authors:** Mauro Iori, Elisa Grassi, Lorenzo Piergallini, Greta Meglioli, Andrea Botti, Giada Sceni, Noemi Cucurachi, Laura Verzellesi, Domenico Finocchiaro, Annibale Versari, Beatrice Fraboni, Federica Fioroni

**Affiliations:** 1 Medical Physics Unit, Azienda USL-IRCCS di Reggio Emilia, 42123 Reggio Emilia, Italy; 2 Department of Physics, University of Bologna, Bologna, Italy; 3 Department of Physics, University of Padova, Padua, Italy; 4 Nuclear Medicine Unit, Azienda USL-IRCCS di Reggio Emilia, Reggio Emilia, Italy

**Keywords:** ^18^F-FDG, PET, SUV, Extravasation, Radiopharmaceutical, Dosimetry

## Abstract

**Background:**

^18^F-FDG PET/CT imaging allows to study oncological patients and their relative diagnosis through the standardised uptake value (SUV) evaluation. During radiopharmaceutical injection, an extravasation event may occur, making the SUV value less accurate and possibly leading to severe tissue damage. The study aimed to propose a new technique to monitor and manage these events, to provide an early evaluation and correction to the estimated SUV value through a SUV correction coefficient.

**Methods:**

A cohort of 70 patients undergoing ^18^F- FDG PET/CT examinations was enrolled. Two portable detectors were secured on the patients' arms. The dose-rate (DR) time curves on the injected DR^in^ and contralateral DR^con^ arm were acquired during the first 10 min of injection. Such data were processed to calculate the parameters Δp^in^_NOR_ = (DR^in^_max_- DR^in^_mean_)/DR^in^_max_ and ΔR_t_ = (DR^in^(t) − DR^con^(t)), where DR^in^_max_ is the maximum DR value, DR^in^_mean_ is the average DR value in the injected arm. OLINDA software allowed dosimetric estimation of the dose in the extravasation region. The estimated residual activity in the extravasation site allowed the evaluation of the SUV's correction value and to define an SUV correction coefficient.

**Results:**

Four cases of extravasations were identified for which ΔR_t_ [(390 ± 26) µSv/h], while ΔR_t_ [(150 ± 22) µSv/h] for abnormal and ΔR_t_ [(24 ± 11) µSv/h] for normal cases. The Δp^in^_NOR_ showed an average value of (0.44 ± 0.05) for extravasation cases and an average value of (0.91 ± 0.06) and (0.77 ± 0.23) in normal and abnormal classes, respectively. The percentage of SUV reduction (SUV_%CR_) ranges between 0.3% and 6%. The calculated self-tissue dose values range from 0.027 to 0.573 Gy, according to the segmentation modality. A similar correlation between the inverse of Δp^in^_NOR_ and the normalised ΔR_t_ with the SUV correction coefficient was found.

**Conclusions:**

The proposed metrics allowed to characterised the extravasation events in the first few minutes after the injection, providing an early SUV correction when necessary. We also assume that the characterisation of the DR-time curve of the injection arm is sufficient for the detection of extravasation events. Further validation of these hypotheses and key metrics is recommended in larger cohorts.

## Background

The European Commission has recently remarked on the importance of the quality and safety of medical applications, which involve ionising radiations, as in the nuclear medicine field where most diagnostic investigations or therapeutic treatments are carried out with radiopharmaceuticals [1]. Their administration procedures are performed mainly via intravenous (IV) injection, and there is interest in making an appropriate clinical service and safeguarding patients treated [2–5].

Although rare, extravasation events can occur during administration due to improper placement of the IV access device or by a failure of the vessel wall [6, 7]. Extravasation should be avoided as the undue absorbed doses in the injection site can alter the result of the study and diagnostic procedures that could be repeated [8], as well as can cause local damage to the patient such as rash or epithelial necrosis, especially in therapeutic treatments. Even if the consequences of extravasation events are considered more dangerous with therapeutic radiopharmaceuticals (beta or alpha emitters) [8], also with diagnostic activity, extravasation can potentially undermine the accuracy of some quantitative parameters affecting clinical diagnosis [9–14]. Radiopharmaceutical extravasation can confound the diagnostic procedure in many ways that include mis- or non-identification of lesions, classifications of scans as non-diagnostic, underestimation of standardised uptake values (SUV) by 19–73%, and the need to repeat imaging with the associated additional radiation exposure [22]. In nuclear medicine, SUV is one of the most important parameters to evaluate the outcome of Positron Emission Tomography (PET) examinations, which are increasingly being used for diagnosis, staging, and therapy response evaluation. Although SUV is characterised by simplicity and ease of use, its measurement is susceptible to many sources of unwanted variability due to physical and biological factors, as well as non-optimised image acquisition, image processing and analysis [9, 10]. Changes in SUV between baseline and follow-up studies can help to determine whether tumours are responding to treatment [11]. Therefore, it is necessary to minimise the sources of error in SUV estimation to produce a semi-quantitative tool as reliable as possible [9, 10]. The SUV accuracy estimation assumes the entire injected dose is administered intravenously, distributing itself completely within the body before the examination is performed; an extravasation event can affect its evaluation. Improper injections can cause extravasations which in turn lower the SUV values, by approximately 10% [14], which in the most serious cases can reach up to 21–50% [11, 12]. From an analysis of 1367 patient, in 18% of studies, there was extravasation with an erroneous accumulation of the injected dose between 1 and 22% [15], while a single-centre analysis highlighted up to 38% of patients with extravasated doses [16].

Over the years, as awareness of avoiding the dose to tissues has grown as well as correctly quantifying the value of the SUV, attention has increased towards extravasations of radiopharmaceuticals. This has been greatly facilitated by the recent commercialisation of a dedicated monitoring system, the LARA device (Lucerno Dynamics, LLC, Cary, NC), able to identify extravasation occurrence. This device employs topically applied scintillation sensors, generally placed on the two arms, to monitor the injected activity (A_in_) process and all the uptake phases whose total duration is of 40–60 min. During the check, a time-activity curve is also plotted and analysed [17–21]. Thanks to this system, it was possible to highlight that the injection of a radiopharmaceutical can exhibit three different behaviours: a correct injection, an abnormal administration, indicative of the patient's venous retention, and an extravasation event. These behaviours can be identified based on the different shapes of their time-activity curves during drug administration [19].

In our work, a new method for monitoring the ^18^F-FDG administration has been developed and proposed, together with new metrics to characterise and classify the administration process, and to provide an early evaluation and correction to the estimated SUV value. As already mentioned, incorrect injections can cause extravasation, which in turn lowers SUV values. Determining a correction coefficient for SUV allows to obtain a correct assessment and use this parameter in clinical practice without using medical imaging to estimate the impact of extravasation. By using two wearables conventional radiation detectors it was possible to effectively monitor the injection process, promptly identify the occurrence of an extravasation event and to associate a SUV correction coefficient. For its wide use in the nuclear medicine field, the focus of our work is on ^18^F-FDG injections, but the same approach can be applied to other radiopharmaceuticals for both diagnostic and therapeutic purposes. Taking as a reference what has been highlighted in the LARA studies, the three expected events that can occur during an administration have been investigated: normal, abnormal and extravasation cases. Compared to what has already been proposed, our system allows to quickly identify the occurrence of an abnormal/extravasation event, with the aim of promptly stopping the injection, and provides an estimate of the correction factor to be applied for an accurate SUV evaluation.

## Materials and methods

### Patient cohort

A total of 70 patients undergoing ^18^F-FDG PET examinations were enrolled in the study approved by our Ethics Committee (approved on 22/06/2021 with Registration number 448/2021/SPER/IRCCSE).

The patients’ age involved in this study were in the range of 22–82 years, with a median of 63 years. Patients without good mobility and/or appropriate clinical conditions, such as not allowing adequate monitoring conditions during the administration of radiopharmaceuticals, were not enrolled in the study. Clinical data such as age, weight, height and glucose level, which could influence the IV procedure, were also acquired to analyse any possible predictions or correlations with abnormal drug administration. Pearson test was used, considered meaningful for a p-value < 0.05, to analyse the existence of some type of correlations with DR-time curves acquisition parameters.

### Monitoring systems

Patient monitoring of IV administration of ^18^F-FDG was performed using a personal spectrometric radiation detector RadEye SPRD-ER of Thermo Fisher Scientific (Waltham, Massachusetts, USA). RadEye SPRD-ER is a high sensitivity gamma radiation detector and DR measurement tool, which incorporates two highly sensitive scintillation detectors, a CsI(TI) working at low dose rates and a PVT (polyvinyl toluene) working at high dose rates, with a miniature photomultiplier for detection of very low radiation levels. It can measure dose in terms of ambient equivalent dose H*(10) [Sv/h] and its sensitivity energy range (40 keV–3 MeV) is suitable for monitoring diagnostic and therapeutic radiopharmaceuticals. It is a wearable device, it could be wireless and it guarantees real-time data transmission through a Bluetooth connection. The instrument is equipped with a display where the detected dose-rate value is shown in real time, and it can also be used as a spectrometer allowing the recognition of radiation sources. Two devices placed on the patient's arms were used for data acquisition. All data were subsequently transferred to the RadEye SPRD-ER v1.47.0 software, which shows both DR-time curves and radiopharmaceutical spectra acquired.

#### ^18^F-FDG injection procedure

**Fig. 1:**
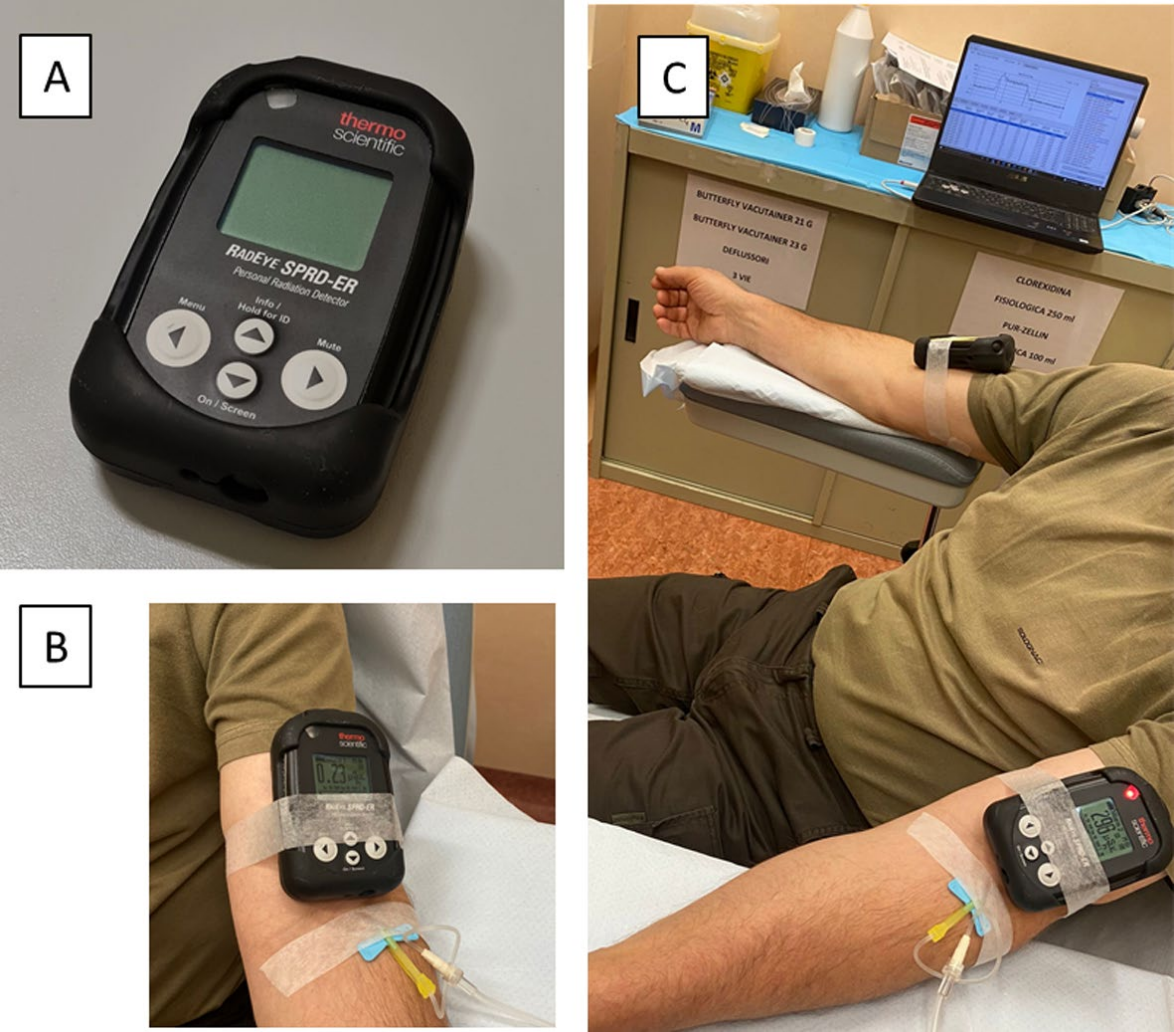
**A** The RadEye SPRD-ER device. **B** RadEye is positioned on the injection arm, which in this case is the left arm. **C** Configuration of signal monitoring procedure during a patient administration by positioning both detectors on the injection (left) and the contralateral (right) arm

**Fig. 2:**
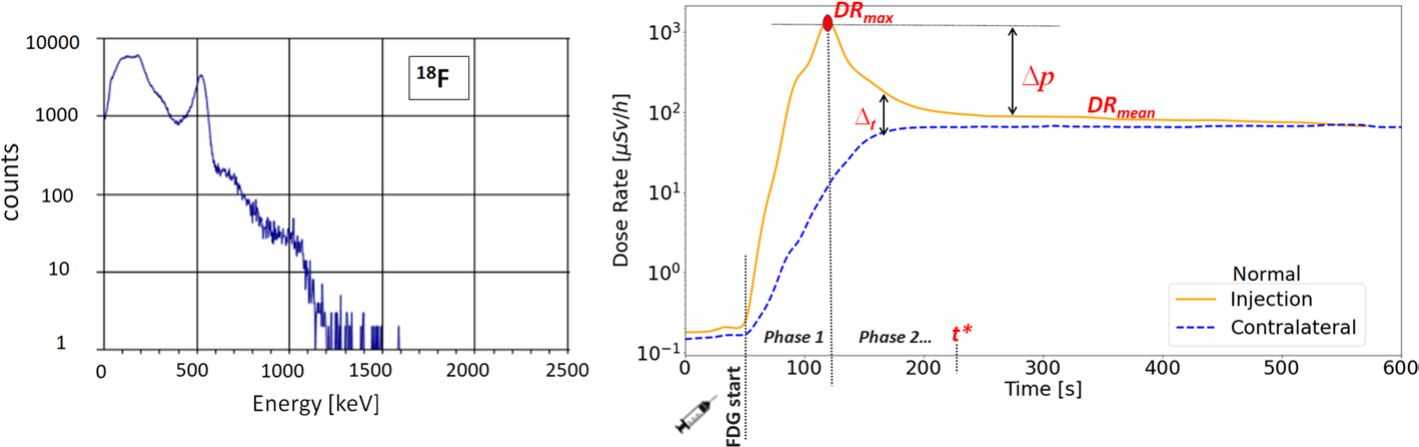
On the left, ^18^F-FDG energy spectrum acquired by the RadEye SPRD-ER device during the initial check. On the right, an illustration of an ideal DR-Time curve. The continuous line is the signal acquired on the injection arm; the dashed line is the contralateral signal

^18^F-FDG injections were performed by nurses with more than 10 years of experience in nuclear medicine field. These procedures consist of two phases: initially, a bolus of drug is injected, lasting an average of 10 s (range 8–12), followed by a second saline infusion of 200 ml, which lasts approximately 7 min. All injections were performed in the ante-cubital fossa. All PET/CT studies were performed on a whole-body PET/CT scanner (Discovery MI, GE Medical System, Milwaukee, WI, USA). Each patient waited a time of 6 h before injection and the examinations were performed 60 min after IV administration of 2.5 MBq/kg of ^18^F-FDG. The blood glucose level measured at the time of the injection was within the acceptable range (i.e. < 200 mg/dl) in all patients. After gaining venous access and before the ^18^F-FDG injection, the two detectors were secured to the patient's arms. The first device was placed approximately 5 cm proximal to the injection site and the second detector was posed on the contralateral arm, making sure that their positions were almost mirrored. The measurement setup is reported in Fig. 1. Both sensors were left in place until the end of the IV administration and all DR data were sent to the control PC. Devices were set for collecting data at 1 s intervals. An example of a generic spectrum of the injected radionuclide and DR-time curve is shown in Fig. 2.

### Injection metric

The DR-time curves acquired during the radiopharmaceutical administration show, on the injection arm, a peak in the first infusion phase that is due to radioactive bolus passage close to the sensor. Reaching a peak, the signal rapidly drops, reaching a plateau level where the DR-time curve of the contralateral arm also converges. In Fig. 2, it is shown the ideal behaviour of a good administration process devoid of abnormal and extravasation events. Based on the literature [17], the ideal trend for a correct IV administration is represented by the convergence towards the plateau of the DR-time curves of both patient arms, considering this behaviour a crucial element for establishing the presence of residual activity (A_RS_) in the injection region. When such behaviour occurs, it is possible to exclude the presence of extravasation. The same assumption was used in this study to evaluate the behaviour of our DR-time curves. Specifically, the following metrics were collected and analysed for each patient, to identify the most suitable parameters to monitor the radiopharmaceutical administration in the first 10 min:

- DR^in^_max_: Maximum DR value acquired by the sensor on the injection arm (in) that corresponds to the passage of radioactive bolus [µSv/h];
- DR^in^_mean_: Average DR value acquired by the sensor on the injection arm after the measured signal reached a plateau [µSv/h];
- t*: Time needed for the DR^in^ value to reach a plateau [sec];
- Δp^in^: It is the difference within the maximum DR value acquired by the sensor on the injection arm and the average DR value acquired by the sensor on the injection arm after the measured signal reached a plateau (DR^in^_max_—DR^in^_mean_);
- ΔR_t_: It is the difference within DR^in^(t) and DR^con^(t), that are the DR values acquired at the same time (t) on injection (in) and contralateral (con) arm, respectively (DR^in^(t) — DR^con^(t)).

### Data analysis

The acquired DR-time curves were analysed, according to the parameters previously described, with a homemade algorithm run manually from a company PC. The algorithm was written in Python v3.7.9 by using the packages of NumPy, Pandas (for building the databases) and Scipy (for the Gaussian filter). The curves were pre-processed through a signal cutting one minute before and nine minutes after the injection peak (DR^in^_max_), which represents the maximum value. The DR-time curves were adapted by Gaussian fit to avoid the noise due to small unavoidable movements of the patient's arm or inevitable movements during the injection.

For each patient, the algorithm identifies values of DR^in^_max_, DR^in^_mean_, t*, and calculates ΔR_t_ and Δp^in^ quantities when the stability of each signal is reached. The condition that must be satisfied to guarantee signal stability is that, for at least 60 consecutive seconds, ΔR_t_ in two consecutive instants (i, i + 1) is not greater than 15 µSv/h. This threshold was established after having evaluated the average values assumed by ΔR_t_ for each consecutive instant on the plateau, considering all administered doses monitored on the enrolled patients. Once the plateau value was reached, evaluations of the patients' DR-time curves were carried out, both by analysing the behaviour of the metric and by defining which of these parameters could identify the expected behaviours for normal, abnormal or extravasation cases. The statistical uncertainties of the devices, corresponding to the relative error on measured DR which is approximately 10%, were also considered.

### Extravasation regions and dose estimations

The A_RS_ in the region surrounding the injection site was estimated from the images using the following method to evaluate its impact on local tissue in terms of dose and to estimate the SUV correction. All the identified extravasation areas were segmented on the PET image and the mean activity concentration values [MBq/ml] were calculated. The process started by determining the extravasation area by manually segmenting the volumes of interest (VOI) choosing a 3-D threshold technique on the PET images using Velocity 3.2 (Varian Medical Systems, Palo Alto, USA).

Different approaches were proposed to segment the volumes most suitable in case of extravasation [11, 23, 24]. Following the strategy proposed by Tylski [23] and Innocent [24], two volumes were segmented corresponding to 10% (Th10) and 40% (Th40) of the maximum intensity uptake voxel values in the extravasated areas. Then, the mean activity concentration values [MBq/ml] in these two volumes were calculated using the Velocity applicative. The total A_RS_ at the injection site was evaluated as the product of the mean activity concentration and the total volume (ml) of the segmented area. Since the identified A_RS_ corresponds to the activity in the extravasation area after 1 h from the administration, the total A_RS_ was decay corrected (physical half-life) by reporting at the time of injection. The dose factors were calculated with OLINDA version 2.3.3 (Hermes Medical Solutions AB, Sweden) using the sphere model [25]. Water spheres of masses compatible with the extravasations were calculated, and their dose per unit of activity was multiplied by the mean activity values to recover the self-dose of infiltrated tissue.

### SUV correction method

The standardised uptake value (SUV) is a semi-quantitative parameter used by nuclear medicine physicians to distinguish between normal and abnormal levels of the tracer uptake. It is well known that SUV has several limitations, and it is dependent on many patient-related factors, such as the VOI definition, the A_in_, the body size, and the time between injection and image acquisition.

PET images of all patients enrolled in the study were analysed, and special attention was paid to all those patients who recorded an anomalous trend of the DR-times curves during administration. PET images were appropriately evaluated by a senior nuclear medicine physician to verify the potential presence of cases of extravasation.

In case of extravasation, the SUV values were corrected by subtracting the estimated activity at the injection area from the value of the total A_in_ [9–11]. In particular, the impact due to extravasation phenomena was quantified.

## Results

### Preliminary data analysis

69 of 70 administrations were considered as one patient was eliminated due to inadequate acquisition conditions. 

Different behaviours in the patient DR-time curves were highlighted by analysing the signals in terms of the parameters ΔR_t_ and Δp^in^. Compared to the entire sample subject of the study, 59 patients reported a normal trend of the DR-time curves, confirmed by PET imaging. Instead, 10 patients showed abnormal trends considering both DR-time curves and their related metrics analysis, whose 4 turned out to be extravasation events as shown by PET imaging. The extracted metrics from the DR-time acquisition curves for the population of cases with normal and abnormal trends were analysed to find possible correlations with the following clinical parameters: age and weight. In none of these cases, a statistically significant correlation was found (all p values were > 0.05).

### Characterisation metrics

Before patient monitoring, the detectors were compared in terms of response using a 100 MBq source of ^18^F, placed inside an injection syringe, and positioned the two detectors at the same distances from the source. In the range of measurements analysed, the detectors showed a variation of the response less than 0.4%. Therefore, no correction factors were used to normalise the readings of the two instruments.

**Fig. 3:**
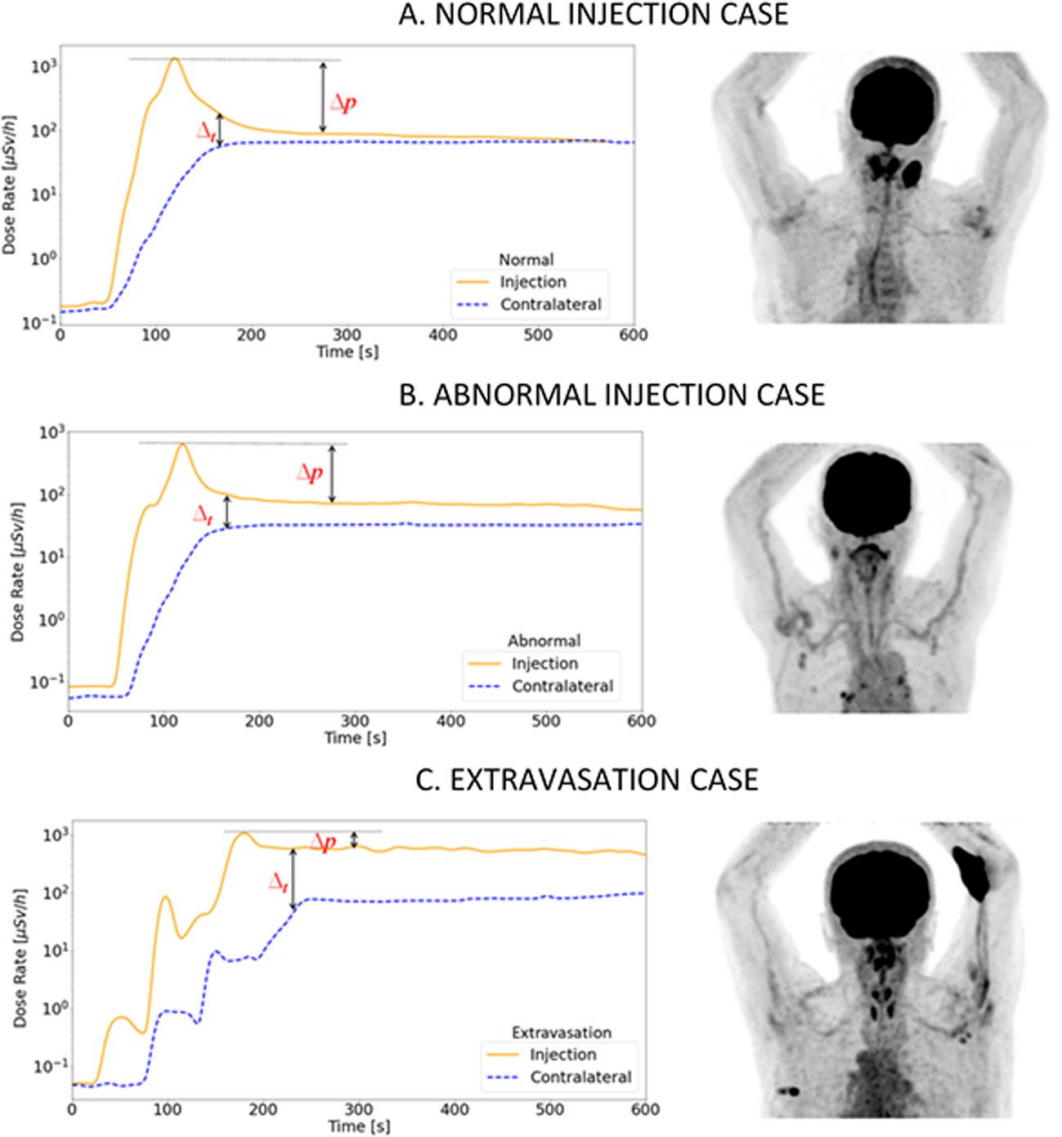
**A** Example of DR-time curves under normal administration together with the corresponding PET image. **B** Example of DR-time curves under abnormal administration together with the corresponding PET image. **C** Example of DR-time curves in case of extravasation together with the corresponding PET image

Different behaviours of DR-time curves are shown in Fig. 3. The curves’ behaviours were characterised by the analysis of the ΔR_t_ and Δp^in^ parameters.

**Fig. 4:**
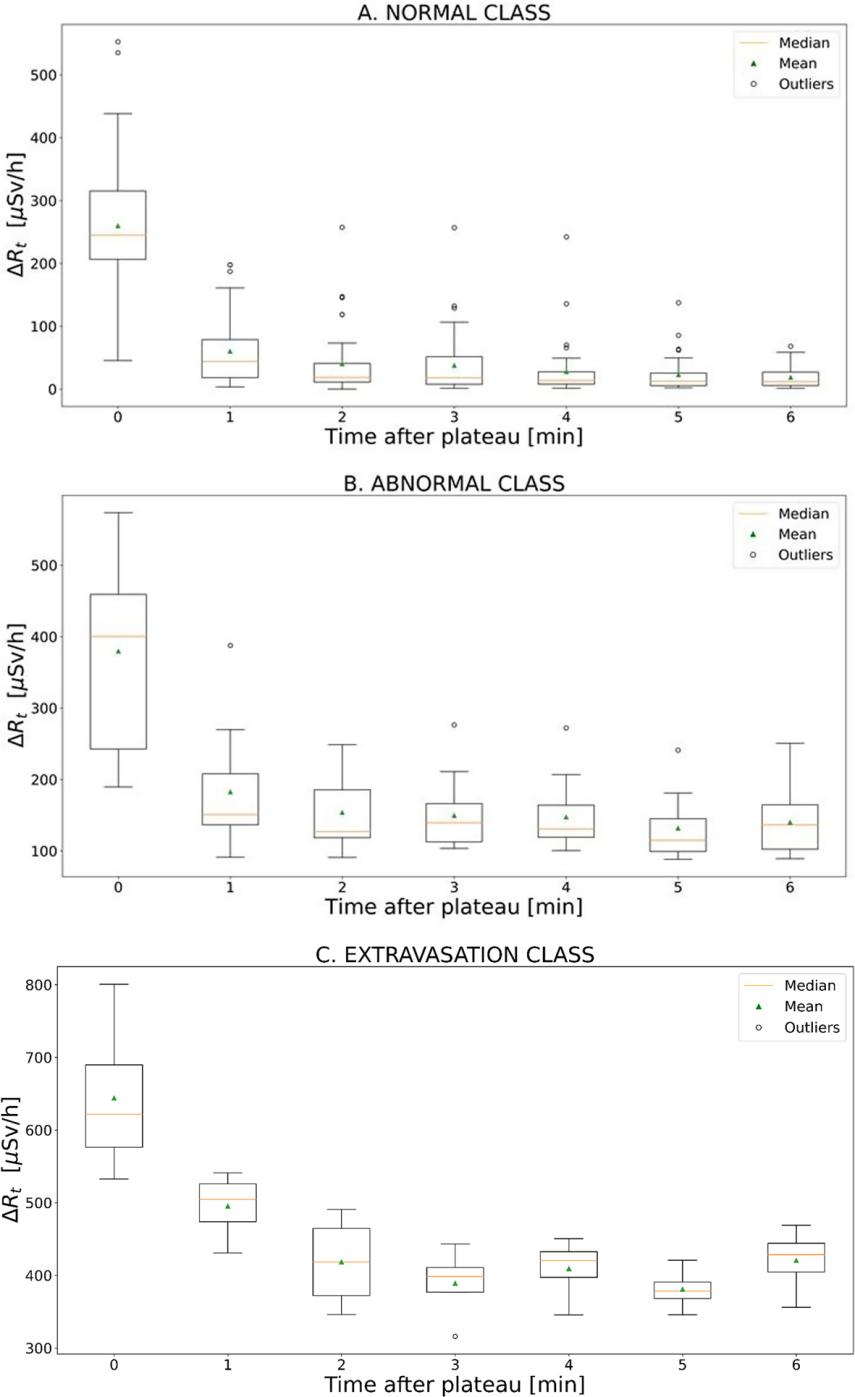
The figure shows the trend of ΔR_t_ with time. **A** Box plot related to the normal administration cases. **B** Box plot related to administration with abnormal behaviour. **C** Box plot related to extravasation cases

Starting from ΔR_t_ analysis, the trend of the ΔR_t_ values of all the acquired cases, setting the plateau start time to time zero, is summarised in Fig. 4. Each boxplot presented in Fig. 4 shows, for any given time interval, the minimum and maximum values, together with the interquartile range and possible outliers. For both normal and abnormal cases, the mean values of ΔR_t_ clearly decrease over time, albeit to a lesser extent for the abnormal case, and even lower extent for the extravasation cases. The analysed patients show, after 6 min from reaching the plateau, an average value of ΔR_t_ for the normal administrations of (24 ± 11) µSv/h, while for an administration with abnormal behaviour, ΔR_t_ is (150 ± 22) µSv/h; for the extravasation ΔR_t_ value is (390 ± 26) µSv/h, showing a decreasing trend not comparable with the normal and abnormal cases. These data are reported in terms of mean value ± 1 standard deviation (SD). A Kruskal–Wallis test was performed to confirm the significant statistical difference between ΔR_t_ of the three classes, after 6 min from reaching the plateau, resulting in a p-value less than 0.05.

**Fig. 5:**
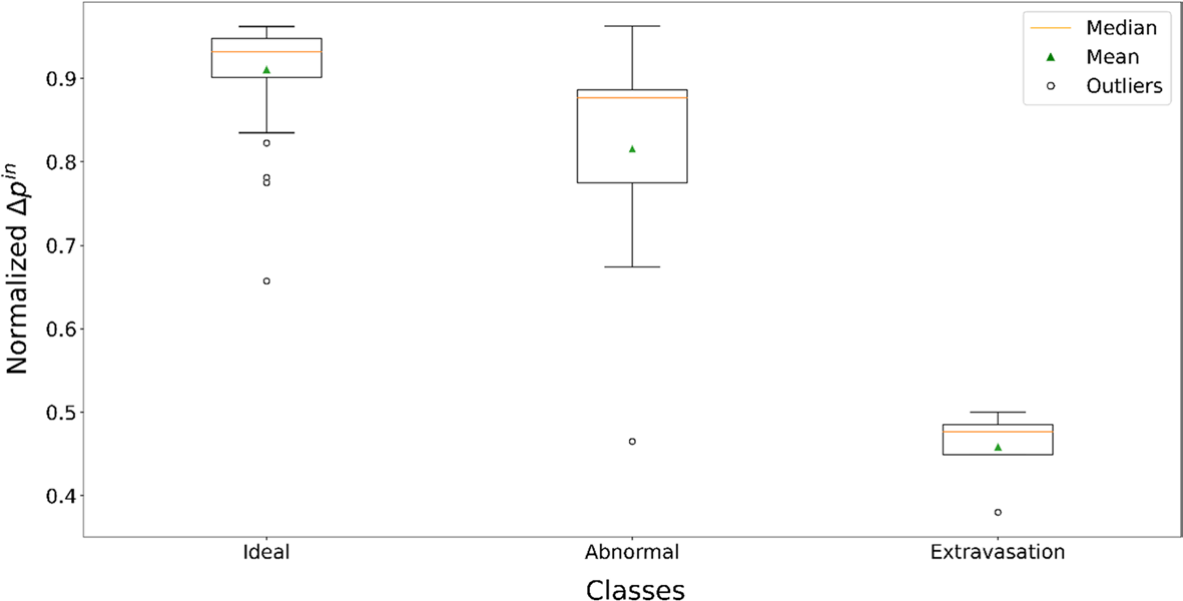
Presentation in boxplot of the Δp^in^_NOR_ values for the normal, abnormal, and extravasation cases

Proceeding with Δp^in^ analysis, the calculation of this parameter is significantly affected by the different activities delivered to the patients as a function of their body weight. So, the Δp^in^ values are required to be normalised. The DR^in^_max_ data were used as factor for their normalisation, providing values Δp^in^_NOR_ = Δp^in^/DR^in^_max_. A completely different trend is obtained for the three administration classes when the values of Δp^in^_NOR_ are analysed. The boxplots of Δp^in^_NOR_ data for the three different types of administration are shown in Fig. 5. The Δp^in^_NOR_ values of normal and abnormal administration values are (0.91 ± 0.06) and (0.77 ± 0.23), respectively, while extravasation falls to (0.44 ± 0.05). These data are reported in terms of mean value ± 1 SD. The Δp^in^_NOR_ values belong to statistically different populations according to the Kruskal–Wallis test (all *p* values are < 0.05).

### Dose estimation and SUV correction calculation

For the extravasation cases, the calculated self-dose in the injection tissue area is shown in Table 1. The table shows the data for the four patients with extravasation, divided according to the type of threshold applied for segmentation.

**Table 1:** Detailed dosimetry results for each extravasation case, including the applied threshold for segmentation and its volume, the average activity concentration (Bq/ml), the A_RS_ in the injection site, A_RS_(Bq), the dose factor (mGy/MBq), and the calculated patients’ self-dose (Gy)

Patient ID	Threshold (%)	Volume (cm^**3**^)	Mean activity concentration (Bq/ml)	A_RS_(Bq)	Dose factor (mGy/MBq)	Self-dose (Gy)
Case 1	Th40	18.6	244,917.7	6,671,178.9	22.4	0.149
	Th10	46.6	142,346.4	9,714,080.8	9.4	0.092
Case 2	Th40	3.7	47,812.4	259,066.5	105	0.027
	Th10	22.8	20,396.3	681,013.4	18.5	0.013
Case 3	Th40	1.4	218,229.5	447,415.4	268	0.120
	Th10	4	117,344.4	687,372.1	97.3	0.067
Case 4	Th40	8	1,570,269.1	18,396,426	50	0.920
	Th10	24.9	924,394.2	33,707,453	17	0.573

To quantify the effect of extravasation events on SUV calculation, the values of A_RS_ at the injection sites were estimated, choosing the threshold at 10% [23] according to the nuclear medicine physician’s opinion. Table 2 shows the A_in_ and A_RS_ reported as a percentage of the activities actually injected in the four extravasation cases. Thanks to these data, it was possible to estimate the corresponding percentage variation of the correct SUV (SUV_%CR_), compared to the values initially estimated without considering the extravasation events (SUV_in_). The four extravasation cases showed significant differences in terms of A_RS_, as well as a wide variation of the SUV_%CR_ (range 0.34–6%). Also, Table 2 reports the Δp^in^_NOR_ values, and ΔR_t_^NOR^ values, which are the ΔR_t_ values, after 6 min from reaching the plateau, normalised to the DR^in^_max_.

**Table 2:** A_in_ (MBq), A_RS_ (%), initially estimated SUV values (SUV_in_) without considering the extravasation events, percentage variation on the correct SUV (SUV_%CR_), Δp^in^_NOR_ and ΔR_t_^NOR^ coefficients, and SUV correction coefficients for the four extravasation cases

Patient ID	A_in_ (MBq)	A_RS_ (%)	SUV_in_	SUV_%CR_	Δp^in^_NOR_	ΔR_t_^NOR^	SUV cor. coeff.
Case 1	163	5.96	1.8	6.334	0.47	0.41	1.063
Case 2	185	0.37	3.2	0.369	0.52	0.33	1.004
Case 3	202	0.34	2.4	0.341	0.58	0.31	1.003
Case 4	165	2.04	2	2.085	0.5	0.38	1.021

**Fig. 6:**
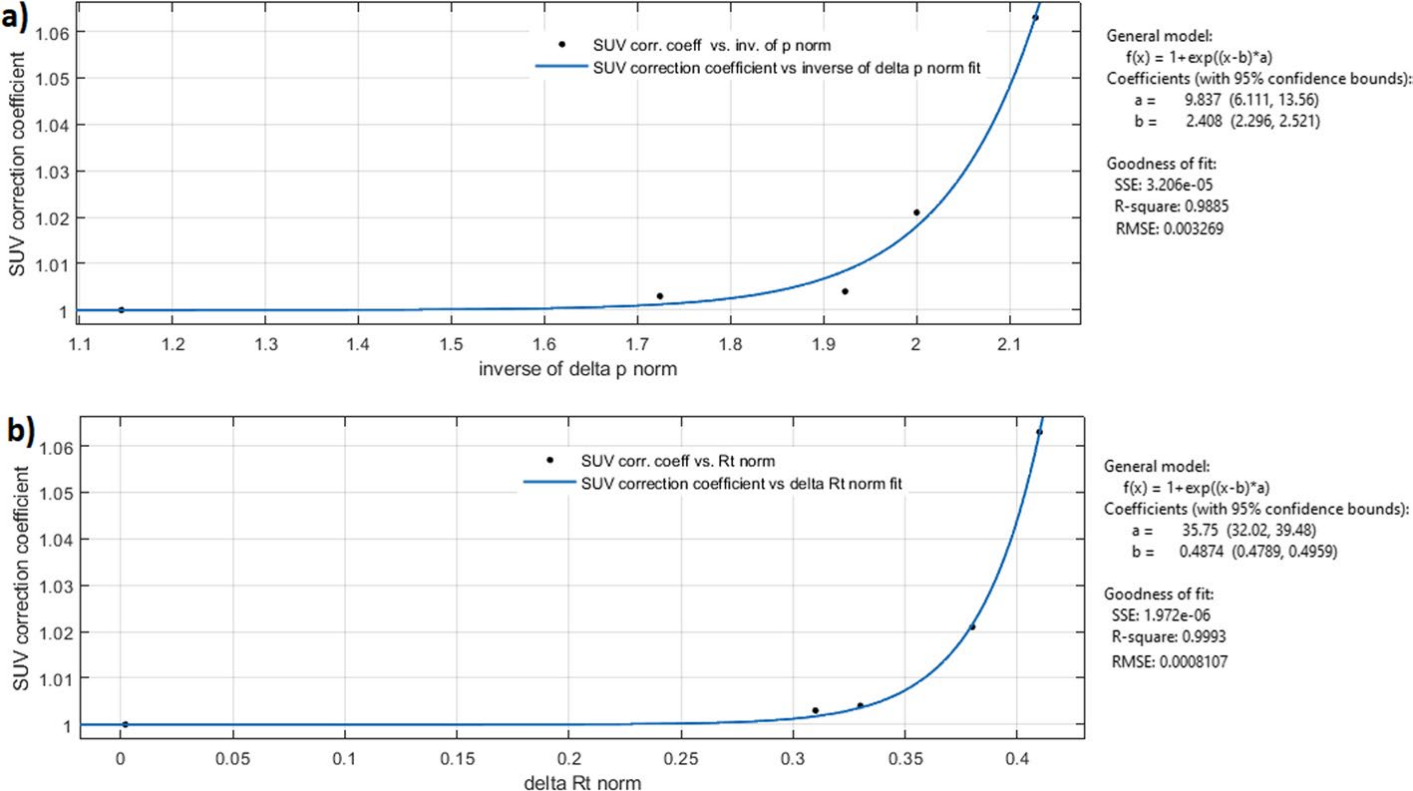
Fitted curves of the inverse of Δp^in^_NOR_ (figure **a**) and ΔR_t_.^NOR^ (figure **b**) versus SUV correction coefficients presented in Table 2. Ideal and abnormal cases were associated with a cumulative point with an SUV correction coefficient equal to 1

The SUV correction factor multiplied by SUV_in_ yields the corrected SUV factor. We put its value to 1 for normal and abnormal cases, while we calculated it as 1 + SUV_%CR_ for extravasation cases. This correction coefficient was used to study the trend of the inverse of Δp^in^_NOR_ and ΔR_t_^NOR^. Figure 6 shows the curves created by placing on the x-axis the value of the metrics, respectively, the ΔR_t_^NOR^ and the inverse of Δp^in^_NOR_, and on the ordinates SUV correction factors. The two graphs and their fittings were created using the Matlab application 'curve fitting'. The fitting function is the same for both metrics:

$${\text{SUV}}\;{\text{correction}}\;{\text{coefficient}} = 1 + e^{{ - a*\left( {{\text{metric}} - b} \right)}}$$

where metric can be ΔR_t_^NOR^ and the inverse of Δp^in^_NOR_, and *a* and *b* are the coefficients of the fitting. Then, were compared the R^2^ factor and the root mean square error (RMSE), which assess the goodness of fit. Both the fittings obtained from the curve inverse of the inverse of Δp^in^_NOR_ vs SUV correction coefficient, and ΔR_t_^NOR^ vs SUV correction coefficient, return an R^2^ equal to 0.99, while the RMSE is 0.003 and 0.0008 for the inverse of Δp^in^_NOR_ and ΔR_t_^NOR^, respectively. To compare the two RMSE was used the Normalised RMSE (NRMSE), calculated by dividing the RMSE for the SD of the distributions of values, resulting in 0.0177 and 0.0175, for the inverse of Δp^in^_NOR_ and ΔR_t_^NOR^, respectively.

## Discussion

This paper presented the feasibility of locally applied portable spectrometers to detect and characterise the presence of anomalies or extravasation events. As far as the authors are aware, this is the first time that, by analysing the signal relating to the first 10 min of radiopharmaceutical administration monitored with two conventional portable spectrometers, a method to correct the SUV value in case of extravasation is proposed. The described metrics could be applied to predict SUV correction factors before PET imaging acquisition. Furthermore, the method indicates the possibility of estimating the presence of extravasation and the need for SUV correction also using a single detector placed on the injection arm.

A commercial device has been proposed on the market, the LARA system, capable of recognising the presence of extravasation, or abnormal retention, by monitoring the patient during the administration phase of the radiopharmaceutical over 40–60 min. LARA sensors record the relative level of radiotracer in the areas of interest during and after administration, they provide clinicians with evidence of the presence of an event of extravasation. Similar information, together with radionuclide identification, can also be obtained with our method which does not use a dedicated commercial system but uses portable spectrometers, which are present and already used in nuclear medicine services. Unlike the publications in the literature that use the LARA system for monitoring [13, 17, 19, 20], we have acquired DR values over a time interval of 10 min instead of 40–60 min. This choice is related to the results obtained in previous works present in the literature that use the LARA device. In each of these works, the acquisitions were made over a time interval between 40 and 60 min, but observing the Time Activity Curves it can be seen that their trend remains almost constant from 10 min onwards. Furthermore, the most significant parameters also used to develop their Deep Learning models, are those related to the first 10 min of acquisitions [20]. The choice of such acquisition times allowed us to detect the presence of anomalous events and provided an early indication of the need for SUV correction for the PET/CT examination. In our patient cohort, 9 out of 69 abnormal administrations were identified. In particular, 4 cases identified as extravasation were confirmed during PET acquisition.

The attention in this work was focused on the trends analysis of the DR-time curves acquired during the administration of 18F-FDG for PET/CT diagnostic examination (Fig. 1). This monocentric study, despite its statistical limitations, allows identifying the trend of the three different classes of administration (i.e. normal, abnormal, extravasation) similar to those already mentioned in the literature [12, 14, 17, 19].

Although DR-time curves of IV administrations showed similar behaviours in terms of DR^in^_max_, DR^in^_mean_, t*, in general three different mean behaviours were noted (Fig. 3), characterised by ΔR_t_ and Δp^in^_NOR_ analysis. The boxplots shown in Fig. 4 highlight how the ΔR_t_ value could be able to identify the three different classes of administration. According to the results obtained, if after 10 min from the start of injection the ΔR_t_ values are still far from the average value indicated for the normal class (24 ± 11 µSv/h), the patient could present an abnormal case of venous retention, or in the worst case, extravasation. The ΔR_t_ values for the three classes are significantly different (*p* < 0.05). As shown in Fig. 5, also the normalised Δp^in^_NOR_ showed a different behaviour for extravasation cases assuming an average value of (0.44 ± 0.05) compared to the average value of (0.91 ± 0.06) and (0.77 ± 0.23) in normal and abnormal classes, respectively. It might help to define a threshold value for both proposed metrics to allow early detection of extravasation events. Using our limited data set, we estimated a threshold value for the two proposed metrics using a logistic function. We associated a dichotomous outcome, 0 in case no extravasation occurred and 1 in the opposite case, for each value of ΔR_t_ and Δp^in^_NOR_. Using the Matlab application “curve fitting”, we plotted the outcome value against the metrics' values and fitted the curve with the logistic function: $\mathrm{outcome}=1/1+{e}^{-k*\left(\mathrm{metric}-a\right)}$, where metric represent the ΔR_t_ or Δp^in^_NOR_ values. The decision limit was set at 0.5, finding that the fitting parameter, ***a***, is the threshold that allows us to discriminate between extravasation events and normal administrations. Specifically, ${a}_{\Delta {p}_{\mathrm{NOR}}^{\mathrm{in}}}=0.61\pm 0.1$ and ${a}_{\left(\Delta {\mathrm{R}}_{\mathrm{t}}\right)}=302\pm 90 \mu Sv/h$. We are aware that the low number of extravasation events (only four) is not enough to reach a statistically significant threshold value. Our future goal is to discriminate with adequate statistical significance a normal administration from an extravasation event. To accomplish this, we determine whether a diagnostic test (e.g. the logistic test) has any ability to discriminate patients with extravasation, defining the appropriate number of patients to involve in the future study. For our purposes, a useful hypothesis on which to base sample size estimation could be whether the AUC exceeds 0.80; the sample size calculation based on large-sample theory for normally distributed data [26] shows that 1000 patients should be enough. This estimation assumes as a null hypothesis that the AUC equals 0.80 vs an alternative hypothesis that the AUC > 0.80 (one-sided test), the expected AUC = 0.9, the positive cases prevalence equal to 0.06, and the type I and type II error rates equal to 0.05 and 0.1, respectively. Indeed, a study with a larger cohort of patients is necessary to obtain a statistically significant threshold, which could allow real-time detection of extravasation events. All the extravasation cases show a percentage variation on SUV that agree with data in the literature, with a maximum percentage variation of 6% [11]. Table 2 presents an inverse proportionality between Δp^in^_NOR_ and the percentage variation of A_RS_ and SUV_%CR_. Instead, it shows a direct proportionality between ΔR_t_ and the percentage variation of A_RS_ and SUV_%CR_. The Δp^in^_NOR_ parameter could intervene in the evaluation of the entity of the phenomenon, returning a priori a percentage directly linked to the A_RS_. Although the analysis of these two parameters requires further statistical support, the extravasation events' behaviour differs considerably from the two other classes identified.

Figure 6 shows the trend of the SUV correction coefficient in function of ΔR_t_^NOR^ and the inverse of Δp^in^_NOR_. Recalling that Δp^in^_NOR_ is a parameter that is calculated on the curve of the injection arm, the fact that the SUV correction factor has the same trend as a function of the inverse of Δp^in^_NOR_ and ΔR_t_^NOR^, makes us assume that it would suffice to acquire only the time-dependent DR curve of the injection arm to characterise the presence of anomalies or extravasation events in real-time. The possibility of recognising and monitoring extravasation events using a single sensor placed in the injection site would also allow us to apply this method in cases where injection occurs in areas different from the antecubital fossa (e.g. in the foot) due to difficulties in accessing the blood vessel. Furthermore, the correlation found between the SUV correction factor and the inverse of Δp^in^_NOR_ makes us assume that from its calculation, the SUV correction can be estimated before image analysis. This method could greatly speed up SUV correction procedures carried out for diagnostic purposes. These results are presented as a preliminary study whose assumptions may be confirmed by a forthcoming study in which more patients will be involved. Together with the trends analysis of the DR-time curves, we investigated tissue self-dose in the injection area due to extravasation of 18F-FDG. We have identified only three works in the literature that were dedicated to dosimetric extravasation dose estimation due to radiopharmaceuticals [22–24]. We believe that the dosimetric characterisation of extravasation events enables clinicians to identify patients at risk of adverse tissue reactions. However, our method has some limitations. First of all, the major source of error in our estimate lies in the choice of using the radiopharmaceutical physical half-time instead of the effective clearance half-time. This choice was made as a precaution, as there was no specific patient curve describing the kinetics of the radiopharmaceutical. In addition, the used dose factors assume to work on a spherical volume and are only an approximation of the segmented anatomical area on the PET images of patients. A voxel-based approach for dose determination with a more refined dose calculation model could be useful to characterise the limits of this approach. Despite these limitations, the dose estimation is consistent with the absence of observable effects, according to the nuclear medicine physician, they aren't serious cases of extravasation given the small percentages of A_RS_.

**Fig. 7:**
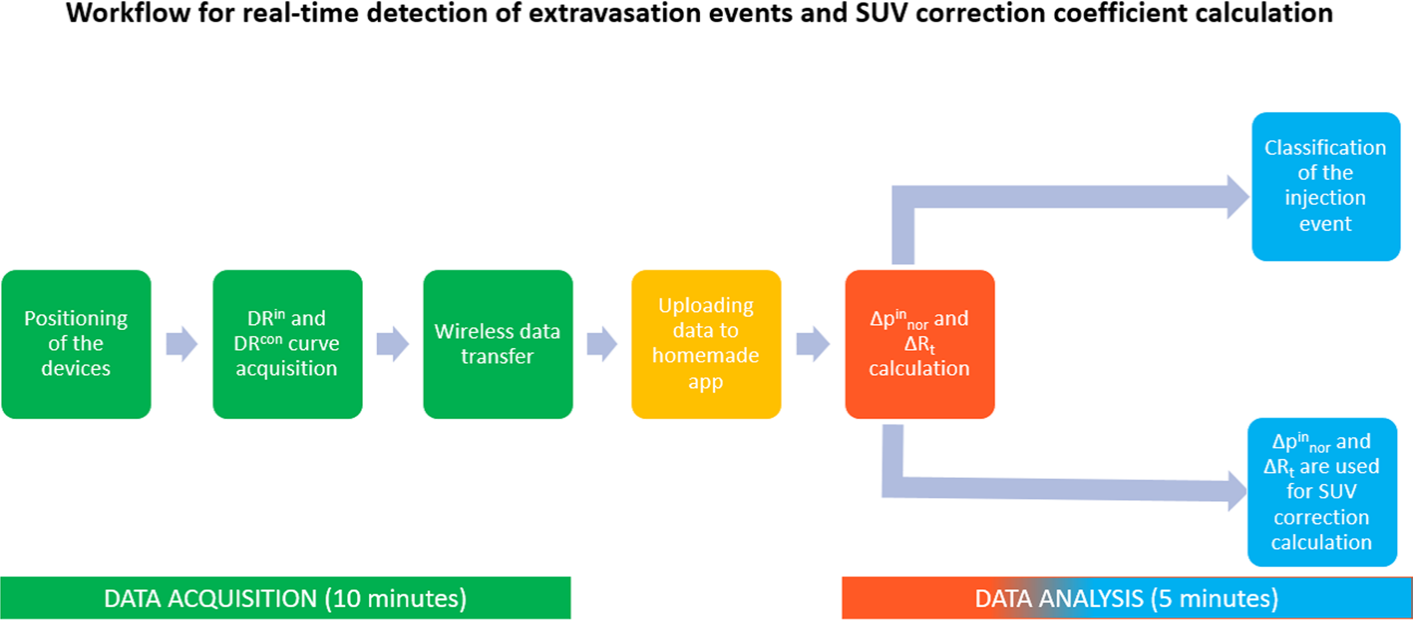
Potential workflow for real-time detection of extravasation events and SUV correction calculation. In round brackets is indicated the time required to complete the two principal steps of the process

The use of the sensors described would make it easier for the healthcare professional in charge of injections to detect extravasations already in the first few minutes after injection. Lightness and great usability encourage further development of this system, which would guarantee a simple method for screening and monitoring IV administration. The real-time detection of DR on the patient’s arms and the reading monitors of devices allows the rapid identification of the ΔR_t_ value, which as described is indicative of identifying abnormal or extravasation cases during injections. We identified a potential workflow for real-time classification of extravasation events and calculation of the SUV correction coefficient, shown in Fig. 7. Furthermore, the proposed monitoring method could also be applied in radiopharmaceutical therapy. It is well known that extravasation episodes during therapeutic treatments can result in non-negligible radiation dose values for the patient. The early detection of these events allows to suspend the radiopharmaceutical infusion, reducing the patient’s risk.

In addition, the characterisation of the Δp^in^_NOR_ parameter may give the opportunity of using only one sensor in the injection zone to characterise extravasation events, as well as allowing the estimation of the SUV correction coefficient.

## Conclusion

The optimisation of the radiopharmaceutical administration process has gained greater attention recently, aiming at timely detecting the extravasation phenomena as well as any other anomalies and to limit their effect. The sensors allowed the collection of dosimetric data directly on patients during administration and allowed an analysis of the different behaviour of DR-time curves. The screening method developed with the metrics proposed can guarantee diagnostic information to the healthcare professional involved in the administration, already in the first minutes after the infusion phase of the drug and it allows early SUV correction coefficient estimation. Further validation of these hypotheses with a larger cohort of patients is necessary to confirm the proposed approach. 

## Data Availability

Data sharing is not applicable to this article.

## Abbreviations

A_in_: Injected activity
A_RS_: Residual activity in the extravasation region
CT: Computed tomography
DR: Dose rate
DR^in^: Dose rate time curve acquired on the injection arm
DR^con^: Dose rate time curve acquired on the contralateral arm
DR^in^_max_: Maximum dose rate value acquired on the injection arm
DR^in^_mean_: Average dose rate value acquired on the injection arm after the measured signal reached a plateau
IV: Intravenous
PET: Positron emission tomography
SD: Standard deviation
SUV: Standardised uptake value
SUV_%CR_: Percentage variation of the correct SUV value
SUV_in_: Initially estimated SUV’s value without considering the extravasation event
t*: Time needed for the DR^in^ value to reach a plateau
VOI: Volume of interest
